# Factors associated with prolonged duration of viral clearance in non-severe SARS-CoV-2 patients in Osaka, Japan

**DOI:** 10.1186/s12199-021-01035-y

**Published:** 2021-12-06

**Authors:** Emma Nakagawa Hoffman, Haruna Kawachi, Atsushi Hirayama, Jingwen Zhang, Ayumi Murayama, Jun Masui, Satomi Fujita, Yasushi Mori, Takanori Hirayama, Toshitake Ohara, Rumiko Asada, Hiroyasu Iso

**Affiliations:** 1 Osaka Prefectural Government, Department of Public Health and Medical Affairs, 1-2 Otemae, Chuo-ku, Osaka, 540-8570 Japan; 2 Public Health, Department of Social Medicine, Graduate School of Medicine, Osaka University, 2-2 Yamadaoka, Suita City, Osaka, 565-0871 Japan

**Keywords:** COVID-19, Virus shedding, Comorbidity, Longitudinal studies

## Abstract

**Background:**

We investigated factors associated with prolonged viral clearance of SARS-CoV-2 among non-severe adult patients in Osaka, Japan. A total of 706 laboratory-confirmed COVID-19 patients were enrolled in this longitudinal observational study between 29 January 2020 and 31 May 2020, across 62 hospitals and three non-hospital recuperation facilities.

**Methods:**

Logistic regression analysis was performed to investigate the factors associated with prolonged (29 days: upper 25% in duration) viral clearance of SARS-CoV-2. Linear regression analysis was conducted to assess these factors 14 days after symptom onset.

**Results:**

The median duration of viral clearance was 22 days from symptom onset. After adjustment for sex, age, symptoms, comorbidity, and location of recuperation, comorbidities were associated with prolonged duration: (OR, 1.77 [95% CI, 1.11–2.82]) for one, (OR, 2.47 [95% CI, 1.32–4.61]) for two or more comorbidities. Viral clearance 14 days after symptom onset was 3 days longer for one comorbidity and 4 days longer for two or more comorbidities compared to clearance when there was no comorbidity.

**Conclusion:**

The presence of comorbidity was a robust factor associated with a longer duration of viral clearance, extending by 3 to 4 days compared to patients with no comorbidity.

**Supplementary Information:**

The online version contains supplementary material available at 10.1186/s12199-021-01035-y.

## Introduction

The outbreak of the coronavirus disease 2019 (COVID-19), induced by severe acute respiratory syndrome coronavirus 2 (SARS-CoV-2) and first discovered in Wuhan City, Hubei Province, China, has rapidly become a global public health emergency [1]. With an estimated case fatality of 2.2% [2], the WHO reported a total of over 232 million confirmed cases and 4.7 million deaths worldwide as of September 2021 [3]. In response to this pandemic, researchers have conducted a wide range of studies concerning the epidemiological and clinical characteristics of SARS-CoV-2, mathematical modeling, and health policy [4–6].

While knowledge has accumulated on the clinical course and outcomes of critically ill patients with COVID-19, research on the viral clearance patterns of non-severe patients is scarce. As new cases continue to surge in Japan, the number of medical emergencies nationwide that required an ambulance dispatch but had difficulty finding a hospital to accept the patient rose for six consecutive weeks to reach a historic high [7]. Hospitals in Osaka have been at maximum capacity for prolonged periods of time, and the lack of hospital beds remain a serious issue. In order to keep hospitals running efficiently, the admission of patients into hospitals are adjusted by the Infected Persons Follow-up Centre of the Osaka Prefectural Government. Currently, patients are admitted depending on severity of disease; however, data analysis to uncover other indicators to adjust patient admission has not been conducted.

Here, we retrospectively analyzed data relating to 706 patients with non-severe, laboratory-confirmed COVID-19 in Osaka and the duration from the onset of disease until consecutive negative detection on reverse transcription polymerase chain reaction (RT-PCR) test of SARS-CoV-2. We examined the duration of viral clearance in mild cases of COVID-19 in order to find out what factors are associated with prolonged viral clearance. These findings can be used as supplementary data when coordinating patients for admission and discharge.

To the best of our knowledge, this is the first study conducted to analyze substantial data from patients who recovered at both hospital and non-hospital facilities in Japan. The viral clearance pattern of SARS-CoV-2 has been investigated in limited studies but can be a useful indicator in coordinating patients for admission and discharge.

## Methods

### Study design and ethical considerations

We conducted a longitudinal observational study on COVID-19 patients in Osaka, Japan. Data from the active epidemiological investigation for COVID-19 under the Infectious Diseases Control Law [8] were used in this study, and data was collected between 29 January 2020 and 31 May 2020. Public health nurses working at public health centres throughout Osaka Prefecture collected the data through active epidemiological investigation, such as patient age, sex, comorbidity, date of onset, symptoms, and PCR testing which was collected by telephone or electronic-based worksheets. Informed consent was not required as data analyses were performed in accordance with the Infectious Diseases Control Law; however, all data were anonymized to uphold confidentiality and patient privacy. This study was approved by the ethical committee of Osaka University (T20114).

### Study population

Osaka Prefecture is in the central area of western Japan and covers an area of 1905 km^2^. Osaka, the third most populous prefecture in Japan, had an estimated population of 8,819,226 as of 1 April 2020 [9]. The first laboratory-confirmed COVID-19 case in Japan was detected on 16 January 2020; thereafter, the first case in Osaka was confirmed on 29 January 2020. Our study population included all laboratory-confirmed COVID-19 patients in Osaka between 29 January 2020 and 31 May 2020; 1783 patients were treated across 62 hospitals and three non-hospital recuperation facilities as well as homes. The last follow-up date was 30 June 2020. All patients were diagnosed via PCR tests on specimens of sputum, nasopharyngeal swabs, or both, according to laboratory guidance from the National Institute of Infectious Diseases, Japan [9].

We restricted patients involved in this study to those who were classified as ‘non-severe’ cases throughout inpatient or non-hospital recuperation. Patient severity was defined by categorization provided by the Japanese Ministry of Health, Labour, and Welfare [10] wherein ‘mild (with respiratory failure)’ indicated difficulty breathing and SpO_2_ levels ≤93% needing oxygen therapy, and ‘severe’ indicated being admitted to the ICU or needing mechanical ventilation. ‘Non-severe’ patients were those who were not categorized in any of these criteria [10]. For asymptomatic patients, the date of specimen collection for a positive test was considered the date of onset, as in the same manner as the guideline principle. As of 31 May 2020, the release criteria for these patients were over 72 h of symptom clearance and two consecutive negative PCR tests on specimens of either sputum or nasopharyngeal swabs, taken 24 h apart. In Osaka, non-severe patients were first admitted to hotel recuperation facilities on 14 April 2020. Admission to non-hospital recuperation facilities was decided by the Infected Persons Follow-up Centre in Osaka Prefecture as requested by the director of public health centres and based on the patients’ vital signs, symptoms, age, and comorbidities. The admission criteria for these patients included non-severe patients, those who maintained independence in activities of daily living (ADLs), and those who had no known active comorbidities requiring medical care.

### Measurements

Clinical characteristics included the following: age, sex, comorbidities (pre-categorized by: diabetes, respiratory disease, coronary heart disease, immunodeficiency, cancer, hypertension, and other), symptoms at the time of admission (pre-categorized by: fever, cough, dyspnea, digestive symptoms, olfactory and taste disorders, fatigue, and other), radiological findings of pneumonia in chest examination of X-ray or CT, location of recuperation, days to admission, and detailed data of PCR testing. The date of symptom onset was defined as the date on which the patients reported the first noticeable symptom. Days to admission was defined as the date from symptom onset to date of admission. Information on comorbidities was self-reported and collected based on interviews with public health nurses. At the time of writing, a definitive treatment for the novel coronavirus was still under investigation. Data on pharmaceutical methods of treatment were not obtained in this study, as all patients were considered non-severe and did not require intensive medical intervention such as intubation or ECMO.

### Outcomes

Duration of viral clearance was calculated as the number of days from the date of symptom onset to the date of specimen collection of the first negative PCR test (out of two consecutive tests). Specimens from nasopharyngeal swabs were used in hospitals for PCR tests; sputum specimens were used to test for negativity at hotels or homes. Prolonged duration of viral clearance was defined as the top quartile of the duration and days after 14 days of symptom onset.

### Statistical analysis

Continuous variables were presented as median and interquartile range (IQR). Categorical variables were presented as numbers and percentages. Comparisons were determined by Mann-Whitney *U* test for continuous variables and chi-square test or Fisher’s exact test for categorical variables as appropriate. Univariate and multivariate logistic regression analyses were performed to explore the association of sex, age groups, location of recuperation, comorbidities, and symptoms with prolonged duration of viral clearance. Prolonged duration of viral clearance was defined as more than 29 days from symptom onset, the top quartile of the overall duration of viral clearance. Logistic regression analysis was used to calculate odds ratios. Linear regression analysis was also performed to examine the days of viral clearance after 14 days of symptom onset, according to each potential predictor. These analyses were stratified by the location of recuperation, that is, hospitals and non-hospitals. Statistical analyses were performed using Stata version 15.1 (StataCorp, College Station, TX, USA). All *p* values were two-tailed with *p* < 0.05 considered statistically significant.

## Results

### Baseline characteristics

**Fig. 1:**
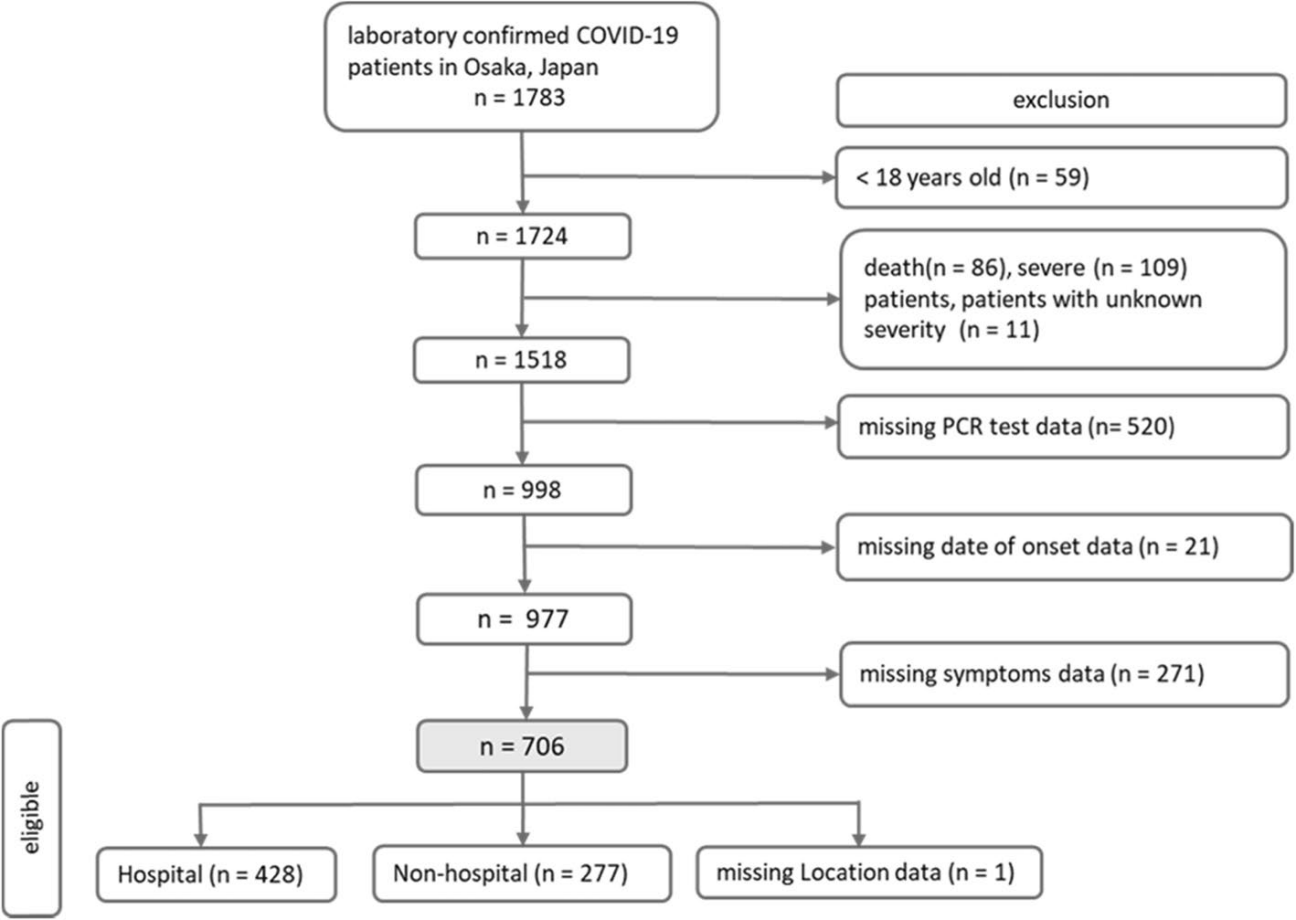
Flow chart of patient selection

This report describes a cohort of 1783 laboratory-confirmed cases of COVID-19 in Osaka. Figure 1 illustrates the patient selection flowchart for this study. A total of 59 patients aged < 18 years were excluded from the study; 109 cases were classified as severe or critical; 86 patients died during the study period; and the severity of 11 cases was unknown and therefore excluded. Cases with missing data on dates of PCR testing (*n* = 520), date of illness onset (*n* = 21), and symptoms (*n* = 271) were also excluded. The total number of cases analyzed in the present study was 706.

The comparison of clinical characteristics is shown in Table 1. Among 706 patients, the median age was 45 years (IQR 31–58); 53.8% were men. Common underlying concomitant diseases included diabetes (7.2%), respiratory disease (7.2%), coronary heart disease (4.5%), cancer (1.6%), and hypertension (1.4%). The major reported symptoms upon admission were fever (> 37 °C; 64.9%), cough (53.4%), and fatigue (38.1%). Upon admission, 33.4% of patients showed signs of pneumonia on radiological chest examination. Of these patients, 60.7% were treated at hospitals and 39.3% were treated at non-hospital facilities.

**Table 1:** Comparison of clinical characteristics between groups by duration of viral clearance

	Overall	Prolonged duration patients	Non-prolonged duration patients	p value
	(*n* = 706)	(*n* = 194)	(*n* = 512)	
Men, *n* (%)	380 (53.8)	107 (55.2)	273 (53.3)	0.66
Age, median [IQR]	45 [31–58]	47 [31–63]	45 [31–57]	0.1
Age categories				
< 30, *n* (%)	140 (19.8)	36 (18.6)	104 (20.3)	0.41
30–39, *n* (%)	144 (20.4)	38 (19.6)	106 (20.7)	
40–49, *n* (%)	130 (18.4)	33 (17.0)	97 (19.0)	
50–59, *n* (%)	131 (18.6)	31 (16.0)	100 (19.5)	
60–69, *n* (%)	55 (7.8)	18 (9.3)	37 (7.2)	
70–79, *n* (%)	57 (8.1)	21 (10.8)	36 (7.0)	
80+, *n* (%)	49 (6.9)	17 (8.8)	32 (6.3)	
Comorbidity, *n* (%)	199 (28.2)	68 (35.1)	131 (25.6)	0.01
Diabetes, *n* (%)	51 (7.2)	19 (9.8)	32 (6.3)	0.1
Respiratory disease, *n* (%)	51 (7.2)	19 (9.8)	32 (6.3)	0.1
Coronary heart disease, *n* (%)	32 (4.5)	12 (6.2)	20 (3.9)	0.19
Immunodeficiency, *n* (%)	8 (1.1)	4 (2.1)	4 (0.8)	0.15
Cancer, *n* (%)	11 (1.6)	3 (1.6)	8 (1.6)	0.99
Hypertension, *n* (%)	10 (1.4)	4 (2.1)	6 (1.2)	0.37
Others, *n* (%)	103 (14.6)	36 (18.6)	67 (13.1)	0.07
Number of comorbidities, *n* (%)				
0	511 (72.4)	126 (65.0)	385 (75.2)	0.02
1	134 (19.0)	44 (22.7)	90 (17.6)	
2+	61 (8.6)	24 (12.4)	37 (7.2)	
Symptoms				
Fever, *n* (%)	458 (64.9)	120 (61.9)	338 (66.0)	0.3
Cough, *n* (%)	377 (53.4)	103 (53.1)	274 (53.5)	0.92
Dyspnea, *n* (%)	164 (23.2)	44 (22.7)	120 (23.4)	0.83
Digestive symptoms, *n* (%)	108 (15.3)	30 (15.5)	78 (15.2)	0.94
Olfactory and taste disorders, *n* (%)	179 (25.4)	50 (25.8)	129 (25.2)	0.88
Fatigue, *n* (%)	269 (38.1)	67 (34.5)	202 (39.5)	0.23
Others, *n* (%)	367 (52.0)	94 (48.5)	273 (53.3)	0.25
Radiological findings (chest examination), *n* (%)	236 (33.4)	65 (33.5)	171 (33.4)	0.95
No findings, *n* (%)	76 (10.8)	22 (11.3)	54 (10.6)	
Missing, *n* (%)	394 (55.8)	107 (55.2)	287 (56.1)	
Duration of viral clearance from symptom onset, median [IQR]	22 [17–29]	34 [31–39]	19 [15–23]	< 0.001
Location of recuperation at the time of the (first) PCR test^a^				
Hospital, *n* (%)	428 (60.7)	100 (51.6)	328 (64.2)	0.002
Non-hospital, *n* (%)	277 (39.3)	94 (48.5)	183 (35.8)	
Hospitalized patients				
Duration from illness onset to admission^b^, median [IQR]	7 [5–10]	10 [7–16]	7 [4–9]	< 0.001
Lengths of hospital stay^c^, median [IQR]	17 (12–23)	28 (22–33)	15 (10–19)	< 0.001

^a^One case (0.1%) had missing data regarding the location of recuperation (*n* = 705)

^b^Six cases (0.8%) had missing data on date of admission (overall *n* = 422; prolonged duration patients *n* = 95; non-prolonged duration patients *n* = 327)

^c^Eight cases (1.1%) of hospitalized patients had missing data on date of admission and/or discharge (overall *n* = 420; prolonged duration patients *n* = 93; non-prolonged duration patients *n* = 327)

**Fig. 2:**
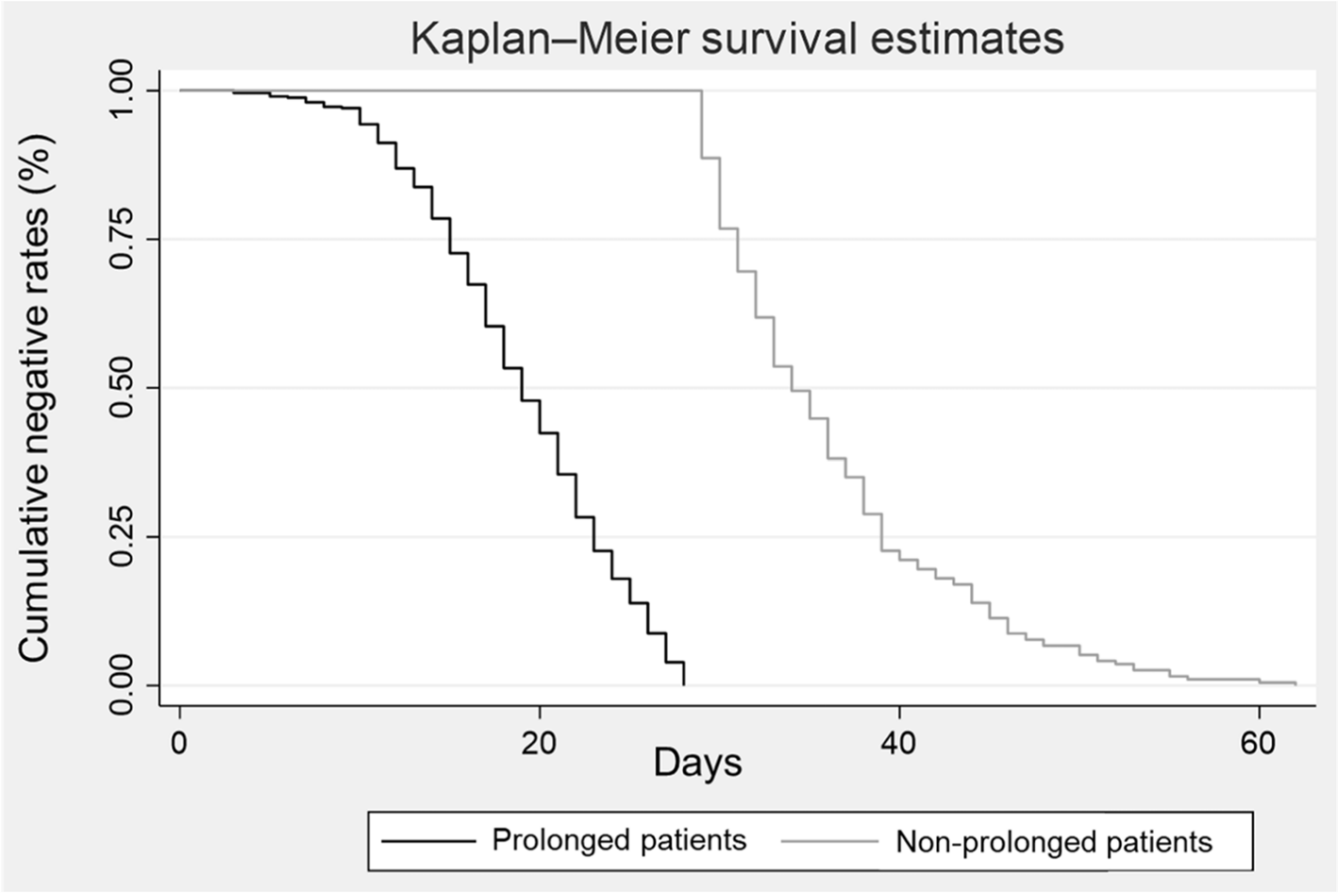
The cumulative rate of viral clearance in prolonged and non-prolonged patients

**Fig. 3:**
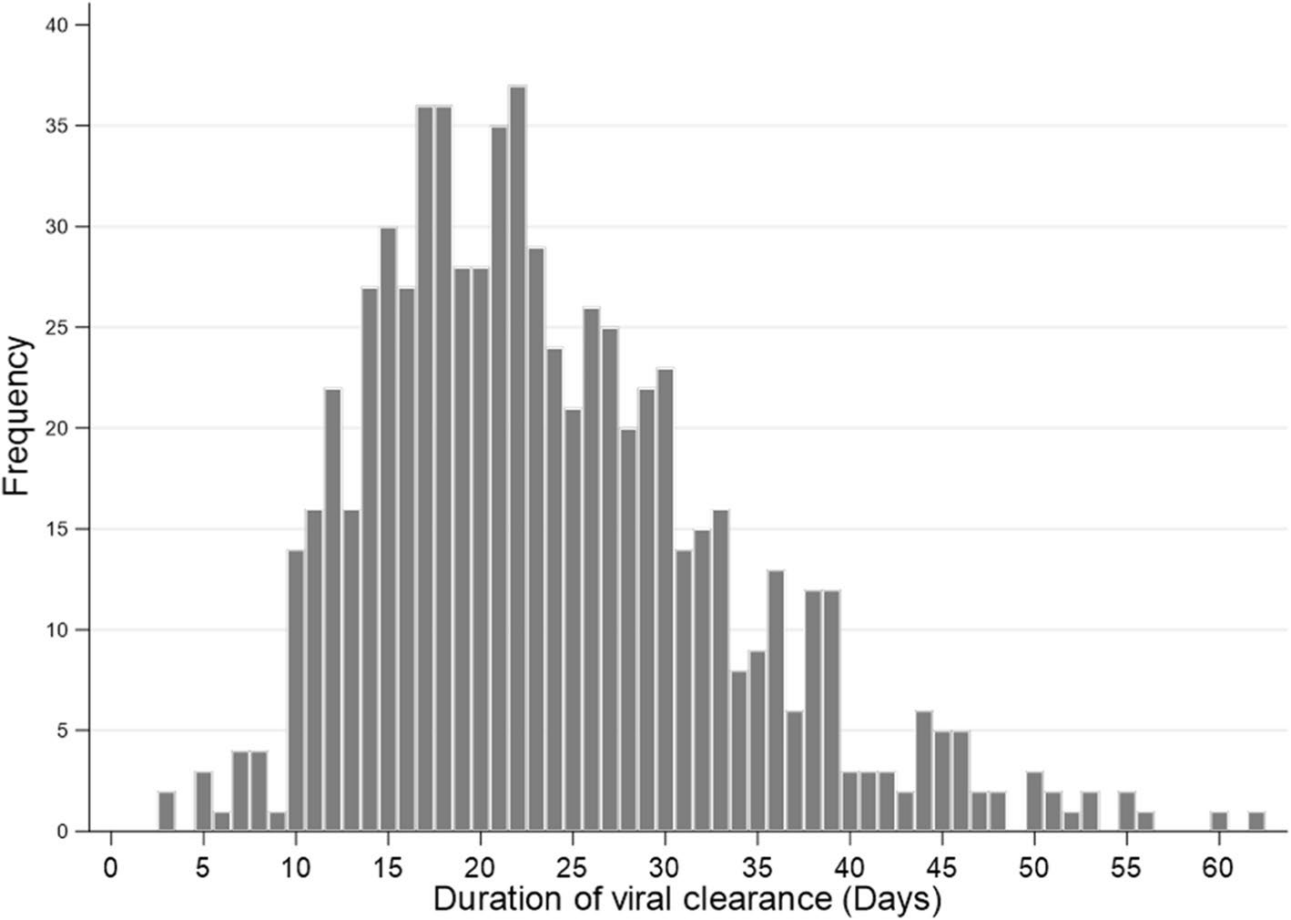
Histogram of the duration of viral clearance

The median duration of viral clearance was estimated to be 22 days (IQR 17–29) in total patients, 34 days (IQR 31–39) in prolonged patients and 19 days (IQR 15–23) in non-prolonged patients (Table 1, Fig. 2). The group with prolonged viral clearance was slightly older (median age 47 years [IQR 31–63]) and had a higher proportion of men (55.2%) than the group without viral clearance, although this was not a significant difference. The prolonged group had a higher number of comorbidities (*p* = 0.02) and a lower proportion of hospital care than the non-prolonged group (51.6% vs. 64.2%, *p* = 0.002). Admission to a treatment facility was delayed in the prolonged group (median days 10 [IQR 7–16]) relative to the non-prolonged group (median days 7 [IQR 4–9]). Figure 3 shows a histogram of the duration of viral clearance among all patients. The overall shape of the distribution is slightly positively skewed to the right. The mode was 22 days, and the maximum value was 62 days. A total of 623 (88%) patients had a viral clearance duration of ≥14 days.

### Logistic regression analysis

The results of univariate and multivariate logistic regression analyses of all patients are presented in Table 2. Both regression analyses were similar for all variables. Multivariate logistic regression analysis indicated that sex and age were not associated with the risk for prolonged duration. Moreover, patients with any comorbidity were associated with risk compared to those without any comorbidity; the risk increased with the number of comorbidities [one comorbidity (OR 1.77 [95% CI 1.11–2.82], *p* = 0.02); two or more comorbidities (OR 2.47 [95% CI 1.32–4.61], *p* = 0.01)]. Patients who recuperated in hospitals presented with a shorter duration of viral clearance (OR, 0.44 [95% CI 0.29–0.65], *p* < 0.001) than those who did not.

**Table 2:** Logistic regression analysis for factors associated with prolonged duration of viral clearance among all patients (*n* = 706)

	Prolonged duration patients (*n* = 194)	Univariate logistic regression			Multivariate logistic regression^a, c^		
Variables (total number (*n*))	*n* (%)	OR	95% CI	*p* value	OR	95% CI	*p* value
Sex							
Women (*n* = 326)	87 (26.7)	ref.			ref.		
Men (*n* = 380)	107 (28. 2)	1.08	0.77–1.5	0.66	1.05	0.74–1.49	0.78
Age: 10 years old increment		1.08	0.99–1.19	0.08	1.09	0.97–1.21	0.13
Comorbidity (*n* = 199)	68 (34.2)	1.57	1.01–2.24	0.01			
Diabetes (*n* = 51)	19 (37.3)	1.63	0.90–2.95	0.11			
Respiratory disease (*n* = 51)	19 (37.3)	1.63	0.90–2.95	0.11			
Coronary heart disease (*n* = 32)	12 (37.5)	1.62	0.78–3.38	0.20			
Immunodeficiency (*n* = 8)	4 (50.0)	2.67	0.66–10.8	0.17			
Cancer (*n* = 11)	3 (27.3)	0.99	0.26–3.77	0.99			
Hypertension (*n* = 10)	4 (40.0)	1.78	0.50–6.36	0.38			
Others (*n* = 103)	36 (35.0)	1.51	0.97–2.36	0.07			
Number of comorbidities							
0 (*n* = 511)	126 (24.7)	ref.			ref.		
1 (*n* = 134)	44 (32.8)	1.49	0.99–2.26	0.06	1.77	1.11–2.82	0.02
2 + (*n* = 61)	24 (39.3)	1.98	1.14–3.44	0.02	2.47	1.32–4.61	0.01
Duration from illness onset to admission (hospitalized patients; *n* = 422)^a^		1.13	1.09–1.17	< 0.001			
Symptoms							
Fever (*n* = 458)	120 (26.2)	0.83	0.59–1.18	0.3	0.96	0.65–1.42	0.85
Cough (*n* = 377)	103 (27.3)	0.98	0.71–1.37	0.92	1.07	0.74–1.53	0.71
Dyspnea (*n* = 164)	44 (26.8)	0.96	0.65–1.42	0.83	1.06	0.69–1.62	0.80
Digestive symptoms (*n* = 108)	30 (27.8)	1.02	0.64–1.61	0.94	1.10	0.68–1.77	0.71
Olfactory and taste disorders (*n* = 179)	50 (27.9)	1.03	0.71–1.51	0.88	1.03	0.69–1.55	0.88
Fatigue (*n* = 269)	67 (24.9)	0.81	0.57–1.14	0.23	0.75	0.51–1.09	0.13
Other (*n* = 367)	94 (25.6)	0.82	0.59–1.15	0.25	0.84	0.59–1.21	0.36
Location of recuperation^b^							
Non-hospital (*n* = 277)	94 (33.9)	ref.			ref.		
Hospital (*n* = 428)	100 (23.4)	0.59	0.42–0.83	0.002	0.44	0.29–0.65	< 0.001

^a^Six cases (1.4%) had missing data on date of admission or location of recuperation. (*n* = 422)

^b^One case (0.1%) had missing data regarding the location of recuperation. (*n* = 705)

^c^Multivariate logistic regression adjusted for sex, age (10 years old increment), number of comorbidities, symptoms (cough, dyspnea, digestive symptoms, olfactory and taste disorders, fatigue, other symptoms), and location of recuperation

*OR* adjusted odds ratio, *95% CI* 95% confidence interval, *ref* reference

The results of the multivariate logistic regression analysis stratified by location are presented in Table 3. Among patients who recuperated in hospitals, increased age was associated with prolonged duration of viral clearance (OR, 1.31 [95% CI, 1.12–1.52], *p* = 0.001). Having two or more comorbidities was also associated with prolonged duration of viral clearance compared to having no comorbidity (OR, 2.74 [95% CI, 1.29–5.83], *p* = 0.01). In addition, the duration from onset of illness to admission was associated with prolonged duration of viral clearance (OR, 1.18 [95% CI, 1.13–1.24], *p* < 0.001). Among patients who recuperated in non-hospital facilities, age and underlying comorbidities were not associated with the risk for prolonged duration of viral clearance.

**Table 3:** Multivariate logistic regression analysis for factors associated with prolonged duration of viral clearance, stratified by location of recuperation

	Hospitalized patients					Non-hospitalized patients				
	Total patients (*n* = 428)	Prolonged duration patients (*n* = 100)	Multivariate logistic regression^a,b^			Total patients (*n* = 277)	Prolonged duration patients (*n* = 94)	Multivariate logistic regression^c^		
Variables	*n* (%)	*n* (%)	OR	95% CI	*p* value	*n* (%)	*n* (%)	OR	95% CI	*p* value
Sex										
Women	188 (43.9)	48 (48.0)	ref.			137 (49.5)	39 (41.5)	ref.		
Men	240 (56.1)	52 (52.0)	0.70	0.42–1.19	0.19	140 (50.5)	55 (58.5)	1.54	0.93–2.57	0.10
Age: 10 years old increment			1.31	1.12–1.52	0.001			0.95	0.78–1.14	0.57
Number of comorbidities										
0	268 (62.6)	49 (49.0)	ref.			243 (87.7)	77 (81.9)	ref.		
1	106 (24.8)	31 (31.0)	1.52	0.81–2.86	0.20	27 (9.8)	13 (13.8)	1.89	0.83–4.29	0.13
2+	54 (12.6)	20 (20.0)	2.74	1.29–5.83	0.01	7 (2.5)	4 (4.3)	3.03	0.64–14.36	0.16
Duration from illness onset to admission^a^			1.18	1.13–1.24	< 0.001					

^a^Six cases (1.4%) of the hospitalized patients had missing data on date of admission. (*n* = 422)

^b^Multivariate logistic regression adjusted for sex, age, number of comorbidities, and duration from illness onset to admission

^c^Multivariate logistic regression adjusted for sex, age and number of comorbidities

*OR* adjusted odds ratio, *95% CI* 95% confidence interval, *ref* reference

### Linear regression analysis

Table 4 summarizes the descriptive statistics and results of the multivariate linear regression analysis examining the factors predicting additional days for viral clearance 14 days after symptom onset. The sample comprised 622 patients (88%) of 706 eligible cases. The results produced corresponded to those of the logistic regression analysis. Men tended to have shorter prolonged days among hospitalized patients and longer prolonged days among non-hospitalized patients. Increased age tended to be associated with longer prolonged days among hospitalized patients and shorter prolonged days among non-hospitalized patients. There was a positive association between the number of comorbidities and prolonged duration of viral clearance among all patients. Patients with one comorbidity experienced 3 days longer duration; those with two or more comorbidities experienced 4 days longer duration when compared to those with no comorbidity (*p* = 0.001 and *p* = 0.002, respectively). For both of the hospitalized and non-hospitalized patients, the prolonged days were slightly shorter among patients with one comorbidity and slightly longer among those with two or more comorbidities, as compared to those with no comorbidity.

**Table 4:** Multivariate linear regression analysis for variables predicting prolonged days of viral clearance 14 days after symptom onset

	All patients (*n* = 622^a^)		Hospitalized patients (*n* = 359^c^)		Non-hospitalized patients (*n* = 257)	
Variables	Adjusted^b^		Adjusted^c, d^		Adjusted^e^	
	β coefficient	*p* value	β coefficient	*p* value	β coefficient	*p* value
Men	0.22	0.76	− 1.53	0.08	2.54	0.01
Age: 10 years old increment	0.04	0.87	0.44	0.08	− 0.56	0.15
Number of comorbidities						
0	ref.	–	ref.	–	ref.	–
1	3.15	0.001	2.98	0.01	2.43	0.15
2+	4.17	0.002	4.76	0.001	4.74	0.17
Recuperation at hospital^a^	− 2.44	0.002	–	–	–	–
Duration from illness onset to admission^d^	–	–	0.6	< 0.001	–	–

^a^One (0.2%) case of all patients had missing data on location of recuperation. (*n* = 622)

^b^Multiple linear regression adjusted for sex, age, number of comorbidities, and location of recuperation

^c^Six (1.6%) cases of hospitalized patients had missing data on date of admission. (*n* = 359)

^d^Multiple linear regression adjusted for sex, age, number of comorbidities, and duration from illness onset to admission

^e^Multiple linear regression adjusted for sex, age and number of comorbidities

## Discussion

We observed that comorbidities were associated with prolonged duration: (OR 1.77 [95% CI, 1.11–2.82]) for one, (OR 2.47 [95% CI, 1.32–4.61]) for two or more comorbidities. Viral clearance 14 days after symptom onset was 3 days longer for one comorbidity and 4 days longer for two or more comorbidities compared to clearance when there was no comorbidity. The number of comorbidities was significantly associated with prolonged duration in a dose-response manner.

An increasing number of studies have illustrated the relationship between COVID-19 comorbidity and disease severity [11–13]. For example, diabetes, a prevalent comorbidity in our study, is characterized by an exaggerated pro-inflammatory cytokine response, notably interleukin-1 (IL), IL-6, and tumor necrosis factor (TNF)-α. Immunological studies suggest that in the absence of appropriate immune stimulation, a cytokine response may be further exaggerated in order to counter the stimulus, as seen in patients with COVID-19 [14, 15]. Additionally, studies on the relationship between infectious diseases and host metabolic processes have speculated that metabolic programming is an essential regulator of inflammatory responses. Physiologic abnormalities, such as type 2 diabetes and hypertension, hinder the recovery of COVID-19 [16].

In our study, delayed hospitalization was associated with prolonged duration of viral clearance. At the time of study, patients could only be discharged from hospitals after testing negative on two consecutive PCR tests. Therefore, delayed hospitalization led to prolonged duration of hospitalization. Although discharge guidelines as of September 2021 do not require consecutive negative PCR tests, delayed hospitalization remains an issue. Ambulance dispatchers have had difficulty finding a hospital to accept the patients and hospitals in Osaka have been at maximum capacity for prolonged periods of time. In order to keep hospitals running efficiently, the findings of this study can be used as supplementary data or used as indicators when adjusting patients for hospitalization.

In our study, the median duration of viral clearance was 22 days from symptom onset. In a Chinese study of 99 patients (median age 54 years [IQR 43–63], 58.4% male), a median SARS-CoV-2 excretion period of 15 days was reported [17]. Furthermore, another Chinese study of 113 patients (median age 51 years [IQR 43–63], 58.4% male) reported a median duration of SARS-CoV-2 RNA detection of 17 days [18]. These Chinese studies were conducted with a retrospective 1-month observation period in hospitals under repeated measurements, regardless of the patient’s symptoms. Additionally, an Italian study of 1162 patients (median age 60.7 [SD 16.3]) reported a median duration of viral clearance of 36 days [19]. In that study, PCRs were tested at 7-day intervals, which may have resulted in a longer duration of viral clearance. Our findings were similar to those of a small Japanese study of 63 patients treated at hospitals in Nara Prefecture (median age 47 [SD 19.9]). This study reported a viral clearance period of 20 days [20].

## Limitations

Our study has several limitations. As this was a multi-centered study, the timing of the negative PCR tests differed between hospitals and hotels. PCR tests for negativity were conducted at frequent intervals in hospitals compared to non-hospital facilities, as more medical staff were available on site. Therefore, the location was stratified; statistical analysis was conducted. This study was conducted in real-world practice and therefore the results of this analysis should be carefully interpreted, as many patients were excluded from the analysis due to missing PCR test results (*n* = 520) and symptom onset (*n* = 271). Supplementary Table 1 shows a comparison of the clinical characteristics between the excluded and non-excluded groups. Compared with the excluded group, the eligible group had a lower proportion of young people under 30 years, a higher proportion of people with comorbidities, and a higher proportion of individuals who recuperated in a hospital. This may have influenced the duration of viral clearance in the eligible group. Furthermore, a limitation of this study was that information on comorbidities was self-reported resulting in the possibility of misclassification. Symptom data was only collected upon admission. Because the duration between onset and admission differed between study subjects, some symptoms may have appeared after admission. Another limitation of this study was the absence of data on viral load. Although the use of sensitive PCR methods offers value from a diagnostic viewpoint, the presence of nucleic acids alone cannot be used to define viral RNA shedding or infectivity. For many viral diseases, such as SARS-CoV and MERS-CoV, it is known that viruses can be detected during testing long after the disappearance of the infectious virus [21, 22]. Wolfel and colleagues reported seroconversion occurred in 50% of patients (out of 14 patients) after 7 days of symptom onset, as live virus could no longer be cultured [23]. At the time of data collection until May 2020, the global standard used for de-isolation and discharge policies was testing negative on two consecutive PCR tests, with each test more than 24 h apart. As knowledge on the infectivity of SARS-CoV-2 has accumulated, global standards will change. The CDC currently recommends the de-isolation of non-severe patients after 10 days if no symptoms are reported [24]. Although viral culture is a beneficial method for evaluating viral infectivity, such data are generally unavailable in clinical practice because of their low sensitivity and long turn-around time for virus detection [25]. Insightful epidemiological analyses may produce sufficient knowledge to improve public health guidelines without the detection of specific agents at the microscopic level.

## Conclusion

Our study presented the patient characteristics and analyzed the viral clearance patterns of non-severe patients who recovered at both hospital and non-hospital facilities. We found that comorbidity was a significant factor associated with prolonged duration of viral clearance, extending by 3 to 4 days compared to when there was no comorbidity. Our findings add to the limited literature on the viral clearance patterns of SARS-CoV-2. Gaining a better understanding of the significance of viral clearance and infectiousness is important for both clinical practice and public policy. Since the effective medicines have not yet been developed for COVID-19, and hospitals continue to operate at maximum capacity, clarifying the viral clearance period for new mutant virus is essential. In this study, we determined underlying comorbidity can be used as an indicator when adjusting patients for hospitalization. What remains critical is incorporating the best evidence of viral clearance and infective risk into routine care practices to reduce excess intervention and fear among healthcare workers and the general population.

## Supplementary Information

**Additional file 1: Supplementary Table 1** Comparison of clinical characteristics between non-severe adult population. **Supplementary Table 2** Comparison of clinical characteristics by location of recuperation

## Data Availability

The data analyzed in this study are not publicly available.

## Abbreviations

COVID-19: Coronavirus disease of 2019
OR: Odds ratio
95% CI: 95% confidence interval
SARS-CoV-2: Severe acute respiratory syndrome coronavirus 2
RT-PCR: Reverse transcription polymerase chain reaction
ADLs: Activities of daily living
IQR: Interquartile range
IL: Interleukin
TNF: Tumor necrosis factor

## Declarations

This study was approved by the ethical committee of Osaka University (T20114).
