# Supplementary material for: Factors associated with prolonged duration of viral clearance in non-severe SARS-CoV-2 patients in Osaka, Japan
Source: Environ Health Prev Med. 2021 Dec 6;26:115. doi: 10.1186/s12199-021-01035-y (PMC8647504; doi:10.1186/s12199-021-01035-y)
Supplement: Supplementary file 1 — Additional file 1: Supplementary Table 1 Comparison of clinical characteristics between non-severe adult population. Supplementary Table 2 Comparison of clinical characteristics by location of recuperation [file 12199_2021_1035_MOESM1_ESM.docx]

Supplementary table 1 Comparison of clinical characteristics between non-severe adult population

|  | Eligible group | Excluded group^a^ |  |
| --- | --- | --- | --- |
|  | (n = 706) | (n = 812) | p value |
| Men, n (%) | 380 (53.8) | 427 (52.6) | 0.63 |
| Age, median [IQR] | 45 [31-58] | 42 [27-57] | 0.001 |
| Age categories |  |  |  |
| <30, n (%) | 140 (19.8) | 237 (29.2) | <0.001 |
| 30-39, n (%) | 144 (20.4) | 138 (17.0) |  |
| 40-49, n (%) | 130 (18.4) | 155 (19.1) |  |
| 50-59, n (%) | 131 (18.6) | 103 (12.7) |  |
| 60-69, n (%) | 55 (7.8) | 69 (8.5) |  |
| 70-79, n (%) | 57 (8.1) | 58 (7.1) |  |
| 80+, n (%) | 49 (6.9) | 52 (6.4) |  |
| Comorbidity, n (%) | 199 (28.2) | 135 (16.6) | <0.001 |
| Diabetes, n (%) |  |  |  |
| yes | 51 (7.2) | 32 (4.0) | 0.01 |
| no | 655 (92.8) | 773 (96.0) |  |
| Respiratory disease, n (%) |  |  |  |
| yes | 51 (7.2) | 35 (4.4) | 0.02 |
| no | 655 (92.8) | 770 (95.7) |  |
| Coronary heart disease, n (%) |  |  |  |
| yes | 32 (4.5) | 23 (2.9) | 0.08 |
| no | 674 (95.5) | 782 (97.1) |  |
| Immunodeficiency, n (%) |  |  |  |
| yes | 8 (1.1) | 5 (0.6) | 0.4 |
| no | 698 (98.9) | 800 (99.4) |  |
| Cancer, n (%) |  |  |  |
| yes | 11 (1.6) | 8 (1.0) | 0.36 |
| no | 695 (98.4) | 797 (99.0) |  |
| Hypertension, n (%) |  |  |  |
| yes | 10 (1.4) | 8 (1.0) | 0.48 |
| no | 696 (98.6) | 797 (99.0) |  |
| Other, n (%) |  |  |  |
| yes | 103 (14.6) | 34 (4.2) | <0.001 |
| no | 603 (85.4) | 771 (95.8) |  |
| Number of comorbidities, n (%) |  |  |  |
| 0 | 511 (72.4) | 657 (80.9) | <0.001 |
| 1 | 134 (19.0) | 104 (12.8) |  |
| 2+ | 61 (8.6) | 51 (6.3) |  |
| Location of recuperation^b^ |  |  |  |
| Hospital, n (%) | 428 (60.7) | 360 (44.8) | <0.001 |
| Non-hospital, n (%) | 277 (39.3) | 444 (55.2) |  |
| ^a^Excluded group due to missing for PCR test, onset date and symptoms. | | | |

Supplementary Table 2 Comparison of clinical characteristics by location of recuperation

|  | Overall | Hospitalized patients | Non-hospitalized patients | p value |
| --- | --- | --- | --- | --- |
|  | (n = 706) | (n = 428) | (n = 277) |  |
| Men, n (%) | 380 (53.8) | 240 (56.1) | 140 (50.5) | 0.15 |
| Age, median [IQR] | 45 [31-58] | 50 [36-68] | 36 [28-50] | ＜0.001 |
| Age categories |  |  |  |  |
| <30, n (%) | 140 (19.8) | 60 (14.0) | 79 (28.5) | ＜0.001 |
| 30-39, n (%) | 144 (20.4) | 70 (16.4) | 74 (26.7) |  |
| 40-49, n (%) | 130 (18.4) | 82 (19.2) | 48 (17.3) |  |
| 50-59, n (%) | 131 (18.6) | 78 (18.2) | 53 (19.1) |  |
| 60-69, n (%) | 55 (7.8) | 37 (8.6) | 18 (6.5) |  |
| 70-79, n (%) | 57 (8.1) | 53 (12.4) | 4 (1.4) |  |
| 80+, n (%) | 49 (6.9) | 48 (11.2) | 1 (0.4) |  |
| Comorbidity, n (%) | 199 (28.2) | 163 (38.1) | 35 (12.6) | ＜0.001 |
| Diabetes, n (%) | 51 (7.2) | 46 (10.8) | 5 (1.8) | ＜0.001 |
| Respiratory disease, n (%) | 51 (7.2) | 38 (8.9) | 12 (4.3) | 0.02 |
| Coronary heart disease, n (%) | 32 (4.5) | 28 (6.5) | 4 (1.4) | 0.001 |
| Immunodeficiency, n (%) | 8 (1.1) | 7 (1.6) | 1 (0.4) | 0.12 |
| Cancer, n (%) | 11 (1.6) | 9 (2.1) | 2 (0.7) | 0.15 |
| Hypertension, n (%) | 10 (1.4) | 9 (2.1) | 1 (0.4) | 0.06 |
| Others, n (%) | 103 (14.6) | 87 (20.3) | 16 (5.8) | ＜0.001 |
| Number of comorbidities, n (%) |  |  |  |  |
| 0 | 511 (72.4) | 268 (62.6) | 243 (87.7) | ＜0.001 |
| 1 | 134 (19.0) | 106 (24.8) | 27 (9.8) |  |
| 2+ | 61 (8.6) | 54 (12.6) | 7 (2.5) |  |
| Symptoms |  |  |  |  |
| Fever, n (%) | 458 (64.9) | 336 (78.5) | 122 (44.0) | ＜0.001 |
| Cough, n (%) | 377 (53.4) | 233 (54.4) | 143 (51.6) | 0.46 |
| Dyspnea, n (%) | 164 (23.2) | 121 (28.3) | 43 (15.5) | ＜0.001 |
| Digestive symptoms, n (%) | 108 (15.3) | 56 (13.1) | 52 (18.8) | 0.04 |
| Olfactory and taste disorders, n (%) | 179 (25.4) | 76 (17.8) | 103 (37.2) | ＜0.001 |
| Fatigue, n (%) | 269 (38.1) | 170 (39.7) | 99 (35.7) | 0.29 |
| Others, n (%) | 367 (52.0) | 208 (48.6) | 159 (57.4) | 0.02 |
| Radiological findings (chest examination), n (%) | 236 (33.4) | 188 (43.9) | 47 (17.0) | ＜0.001 |
| No findings, n (%) | 76 (10.8) | 45 (10.5) | 31 (11.2) |  |
| missing, n (%) | 394 (55.8) | 195 (45.6) | 199 (71.8) |  |
| Duration of viral clearance, median [IQR] | 22 [17-29] | 21 [16-28] | 24 [18-31] | ＜0.001 |
